# Er:YAG Laser Energy Optimization for Reducing Single-Species Microbial Growth on Agar Surfaces In Vitro

**DOI:** 10.3390/pathogens14121287

**Published:** 2025-12-14

**Authors:** Jakub Fiegler-Rudol, Małgorzata Kępa, Dariusz Skaba, Rafał Wiench

**Affiliations:** 1 Department of Periodontal and Oral Mucosa Diseases, Faculty of Medical Sciences in Zabrze, Medical University of Silesia, 40-055 Katowice, Poland; 2 Department of Microbiology, Faculty of Pharmaceutical Sciences in Sosnowiec, Medical University of Silesia, 41-200 Sosnowiec, Poland; mkepa@sum.edu.pl

**Keywords:** agar, *Candida albicans*, *Enterococcus faecalis*, *Escherichia coli*, in vitro, laser therapy, lasers, *Pseudomonas aeruginosa*, *Staphylococcus aureus*

## Abstract

Background: Standardized Er:YAG laser settings for microbial reduction remain undefined, and existing studies rarely compare multiple species under identical conditions. This work aimed to characterize susceptibility across selected microorganisms using a controlled agar-based surface growth model. Methods: Six reference strains (*E. coli*, *S. aureus MSSA*, *S. aureus MRSA*, *E. faecalis*, *P. aeruginosa*, and *C. albicans*) were cultured on agar and exposed to Er:YAG irradiation. Two experimental phases were conducted: (1) inhibition zone mapping using energies between 30 and 400 mJ at 1 Hz, with tapered and flat laser tips; and (2) quantification of viable surface coverage after irradiating mature 96 h cultures with 80, 130, 180, and 230 mJ at 10 Hz in contact mode. ImageJ analysis was used to measure inhibition diameters and remaining coverage. Data were evaluated using two-way ANOVA. Results: All microorganisms showed measurable inhibition at every tested energy level, with diameter increasing proportionally to energy. *E. coli* and *E. faecalis* produced the largest inhibition zones in the mapping phase, while *P. aeruginosa* and *C. albicans* required higher energies to reach comparable levels. Mature surface cultures showed progressive reductions in viable coverage; the strongest effects occurred at 230 mJ. The tapered tip generated broader inhibition zones at lower energies compared with the flat tip. Conclusions: Er:YAG laser irradiation produces consistent, energy-dependent antimicrobial effects on single-species agar-based surface growth, with clear differences in species susceptibility and tip performance. The identified parameter ranges provide a quantitative foundation for future in vitro studies aiming to refine Er:YAG-based microbial reduction strategies.

## 1. Introduction

### 1.1. Background

Microorganisms readily form dense, surface-associated communities that display elevated tolerance to antimicrobial agents. This tolerance arises from restricted penetration of disinfectants, cooperative metabolic interactions, and protective extracellular matrices [1,2,3,4,5,6,7,8,9]. Several organisms are frequently used to model these behaviors in laboratory studies. *Staphylococcus aureus* is a common microorganism for examining surface colonization and antimicrobial tolerance. *Enterococcus faecalis* is notable for its ability to survive harsh environmental conditions. *Pseudomonas aeruginosa* produces a robust, polysaccharide-rich phenotype linked to high resilience. *Escherichia coli* serves as a fast-growing reference species with well-characterized stress responses. *Candida albicans* forms dense fungal structures with physiological properties distinct from bacteria [10,11,12,13,14,15]. The bactericidal activity of the Er:YAG laser is driven largely by its strong absorption by water at 2.94 µm, which leads to rapid vaporization of intra- and extracellular water and the generation of localized pressure waves that disrupt cells on hydrated surfaces [15,16,17,18,19]. The combined photothermal and photomechanical effects yield microexplosive ablation capable of removing or damaging surface-associated microorganisms [17,18]. In hydrated substrates such as soft tissues, dentin, or laboratory media, this mechanism supports material removal with limited thermal diffusion to adjacent structures when pulse energy, duration, and repetition rate are properly controlled [19,20,21,22,23,24,25,26,27,28,29].

### 1.2. Rationale

Previous in vitro studies have shown reductions in microbial load across a variety of substrates, although reported protocols differ widely in energy settings, exposure durations, and delivery geometries [19,20,21,22,23,24,25,26,27,28,29], and a critical remaining gap is the limited understanding of species-specific responses under rigorously standardized conditions, as differences in surface layer density, composition, and stress tolerance among organisms likely influence susceptibility to Er:YAG exposure [20,25,30,31,32], making single-species models essential for establishing reference energy thresholds and identifying microorganisms that require higher or repeated irradiation [20,21,22,23,24], especially since Er:YAG parameters reported in the literature vary widely and most studies investigate only one or two microorganisms at a time, limiting species-specific comparability under standardized conditions [20,21,22,23,24], and few investigations have systematically mapped how graded energy settings influence the reduction in surface-associated microbial growth when experimental conditions are kept constant, which motivated the present in vitro optimization study using single-species cultures on hydrated agar to characterize energy-dependent responses across a diverse microbial panel, identify pulse energies that consistently produce measurable inhibition zones and reductions in viable surface coverage, and generate microorganism- and energy-specific reference data that provide a controlled framework for understanding Er:YAG interactions and establishing baseline parameters for future in vitro research using more complex models or alternative substrates.

## 2. Materials and Methods

### 2.1. Study Design

This in vitro study evaluated the effects of Er:YAG laser irradiation on eradicating single-species microbial surface growth established on hydrated agar. The experiment consisted of two phases:

1. Phase I: Mapping inhibition zone formation at graded energy levels.
2. Phase II: Assessing reduction in viable surface coverage in mature agar-based cultures.

The independent variable was the laser energy level, and the dependent variables were inhibition zone diameter and viable culture coverage. Each condition in Phase I was tested four times, and each condition in Phase II was tested three times. Sample size was based on preliminary trials designed to detect statistically meaningful differences in microbial reduction.

### 2.2. Null Hypothesis

The null hypothesis stated that laser energy level and microbial species would have no significant effect on inhibition zone diameter or viable microbial growth coverage.

### 2.3. Objectives

The primary objective of this study was to determine the optimal Er:YAG laser parameters for effective disruption and inhibition of single-species microbial growth. Specific aims included:

- To measure the relationship between laser energy and inhibition zone diameter across different microbial species.
- To quantify the reduction in viable microbial growth at defined laser energy settings.
- To compare the performance of tapered and flat laser tips in energy delivery and antimicrobial effectiveness.

### 2.4. Bacterial and Fungal Strains

Reference strains were obtained from the American Type Culture Collection (ATCC, Manassas, VA, USA): *Escherichia coli* ATCC 25922, *Staphylococcus aureus* ATCC 43300—MRSA (*methicyllin-resistant Staphylococcus aureus*) and *Staphylococcus aureus* ATCC 25923—MSSA (*methicyllin-sensitive Staphylococcus aureus*), *Enterococcus faecalis* ATCC 29212, *Pseudomonas aeruginosa* ATCC 27853, and *Candida albicans* ATCC 10231.

All strains were sourced from the strain bank of the Department of Microbiology, Faculty of Pharmaceutical Sciences in Sosnowiec, Medical University of Silesia in Katowice, and stored at −80 °C in tryptic soy broth containing glycerol. Selection and sample size were guided by previous studies on Er:YAG laser antimicrobial activity to ensure reproducibility and statistical reliability [20,21,22,23,24].

### 2.5. Cultivation Conditions

Each bacterial strain was subcultured on tryptic soy agar (TSA), and the single fungal strain was cultured on Sabouraud Dextrose Agar (SDA) to verify viability and purity. Cultures were incubated at 37 °C under aerobic conditions for 24 h. Colonies were then suspended in 0.9% sodium chloride (NaCl), and cell density was standardized to the 0.5 McFarland scale using a DensiLaMeter II spectrophotometer (Erba Lachema, Brno, Czech Republic) at 525 nm. An inoculum with a density of 0.5 on the McFarland scale corresponds to about 1 to 2 × 10^8^ CFU/mL for bacteria and 1 to 5 × 10^6^ CFU/mL for fungi. Where needed, suspensions were subsequently diluted to the target inoculum for plating.

### 2.6. Study Groups

Fresh 100 µL aliquots of the standardized microbial suspensions were evenly spread on agar plates. Twelve Petri dishes were prepared in total, with two assigned per strain. This design allowed for direct comparison of the antimicrobial effects of laser exposure.

### 2.7. Assessment of the Antimicrobial Effectiveness of Er:YAG Laser Irradiation on Single-Species Culture Growth

#### 2.7.1. Agar-Based Microbial Surface Layer

Five bacterial species, *Staphylococcus aureus* (ATCC 25923 and ATCC 43300), *Pseudomonas aeruginosa* (ATCC 27853), *Enterococcus faecalis* (ATCC 29212), and *Escherichia coli* (ATCC 25922), were cultured on TSA, and *Candida albicans* (ATCC 10231) was cultured on SDA, depending on microbial type. Each inoculated plate was incubated for 6 h at 37 °C to allow the formation of an agar-based microbial surface layer prior to laser exposure.

#### 2.7.2. Laser Irradiation Protocol

A custom-designed template containing 32 equidistant exposure points arranged in two concentric circles (20 mm apart) was placed beneath each Petri dish to ensure consistent irradiation geometry. Laser exposure was performed using an AdvErL Evo Er:YAG laser system (J. Morita, Osaka, Japan) in single-pulse mode at 1 Hz frequency with a pulse duration of 300 µs. The PS600T laser tip (tapered, diameter 0.6 mm; effective area ≈ 0.0028 cm^2^) or C600F (flat, diameter 0.6 mm; area ≈ 0.0028 cm^2^) was used without air or water cooling to allow direct laser–culture interaction.

**Figure 1:**
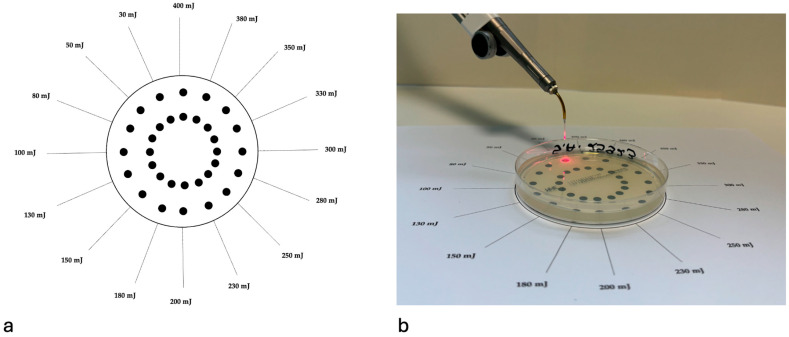
(**a**) Template Design for Er:YAG Laser Irradiation Showing Energy Distribution Across 32 Target Points. (**b**) The experimental setup involved a Petri dish filled with agar. A 100 µL suspension was applied onto a template featuring 32 marked points, each representing a target site for Er:YAG laser exposure (AdverEvo, Morita, Osaka, Japan).

Average output power was 0.03–0.40 W at 1 Hz. Pulse peak power was 100–1333.33 W (E/τ = 30–400 mJ over 300 µs), giving peak power density of 35,714.28–476,189.28 W/cm^2^, fluence of 0.0032–0.043 J/cm^2^. The laser handpiece was held in a fixed vertical position with a mechanical holder to maintain a constant 10 mm distance from the agar surface. Each microorganism–energy combination was tested four times. This is shown in Figure 1.

#### 2.7.3. Incubation and Imaging

After irradiation, the plates were incubated aerobically at 37 °C for 48 h. Post-incubation, high-resolution digital images were taken using a Nikon D70s DSLR camera (Nikon Corporation, Tokyo, Japan) equipped with an 18–70 mm f/3.5–4.5 NIKKOR lens. All images were captured under standardized conditions, fixed illumination, exposure, and a 34 cm shooting distance to ensure reproducibility and accurate measurement of inhibition zones.

#### 2.7.4. Quantitative Analysis

Quantitative assessment of microbial inhibition was performed using ImageJ-Fiji software (version 1.53j; National Institutes of Health, Bethesda, MD, USA) (last use: 3 November 2025). The diameter of the growth inhibition zone was measured in millimeters after 24 h and re-evaluated at 48, 72, and 96 h to track time-dependent changes. Measurements were taken along the central axis of each inhibition zone and calibrated using the software’s internal scale. Data were compiled in Microsoft Excel (Microsoft Corporation, Redmond, WA, USA) and subjected to statistical analysis to determine the relationship between laser energy, microbial species, type of laser tip, and inhibition zone diameter.

### 2.8. Assessment of the Er:YAG Laser’s Effectiveness in Removing Mature Single-Species Cultures

#### 2.8.1. Culture Maturation

The second phase evaluated the effect of Er:YAG laser irradiation on mature single-species cultures. Each microorganism was grown on the appropriate agar medium, SDA for *Candida albicans* and TSA for all bacterial strains, and incubated aerobically at 37 °C for 96 h. This incubation period ensured the formation of stable, confluent surface growth suitable for irradiation.

#### 2.8.2. Laser Irradiation Procedure

**Figure 2:**
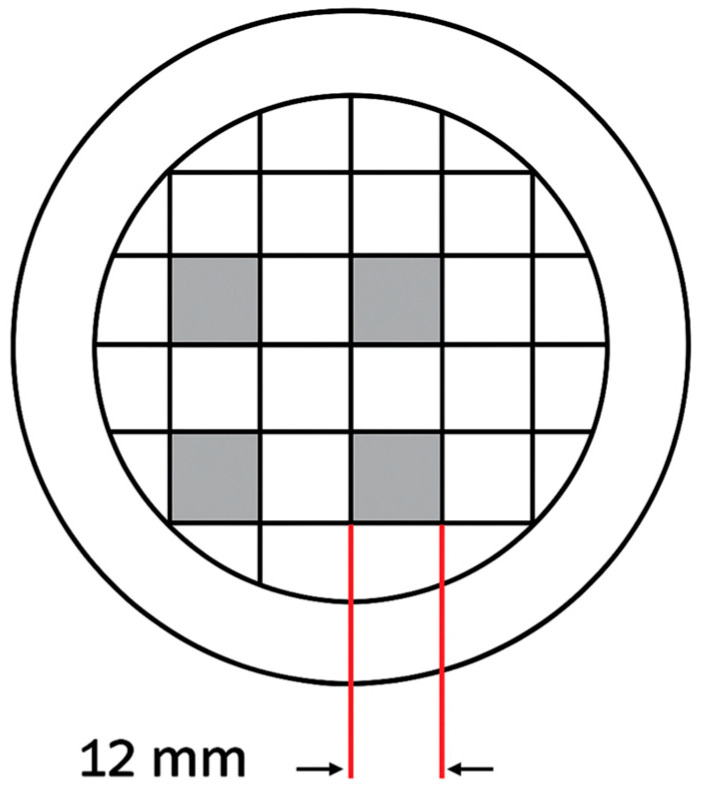
Schematic of the irradiated grid showing the selected squares on the agar plates. The top left square was irradiated with 80 mJ, the top right with 130 mJ, the bottom left with 180 mJ, and the bottom right with 230 mJ.

A pre-designed template (Figure 2) was positioned beneath each Petri dish to define four square irradiation zones (12 × 12 mm), each representing a distinct target area. Laser exposure was performed using the AdvErL Evo Er:YAG system (J. Morita, Osaka, Japan) operating in pulse mode at a frequency of 10 Hz and pulse duration of 300 µs. The C600F laser tip (diameter 0.6 mm; area ≈ 0.0028 cm^2^) was used in contact mode at approximately a 45° angle to the plate surface.

**Figure 3:**
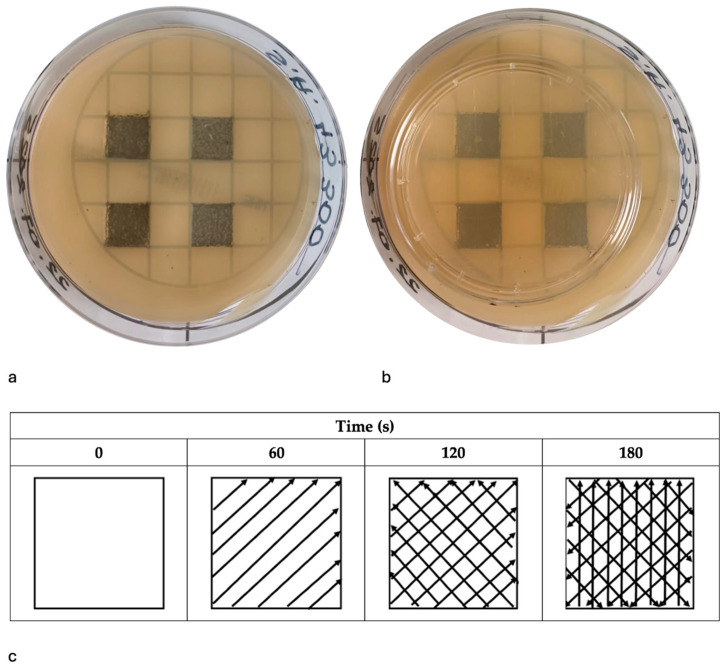
(**a**) Agar plate showing the four predefined square irradiation zones (12 × 12 mm) after laser application. (**b**) Sampling using Rodac IRR LAB-Agar contact plates (**c**) Schematic representation of the irradiation pattern within a single square over time. At 0 s the area is untreated. At 60 s the laser tip has passed over the surface in one diagonal direction, at 120 s a second diagonal pass is added to create a crosshatch pattern, and at 180 s vertical passes are added.

Figure 3c shows the laser irradiation procedure, which lasted 180 s in total. These parameters were selected based on results from the inhibition phase, where lower energies produced detectable antimicrobial effects, allowing systematic evaluation of dose-dependent removal of the agar-based microbial surface layer. Average output power was 0.8–2.3 W at 10 Hz, while pulse peak power was 267–767 W (yielding the peak power densities above). Peak power density was 95,238.09–273,809.5 W/cm^2^.

No air or water cooling or aiming beam was applied to maintain direct photothermal and photomechanical interaction between the laser and the culture surface. Irradiation followed a consistent sequence, starting with the upper left quadrant (80 mJ) and proceeding clockwise through 130, 180, and 230 mJ. The laser tip was cleaned with sterile gauze every 60 s to prevent debris buildup; cleaning time was not included in the exposure duration.

#### 2.8.3. Sampling and Incubation

Immediately after irradiation, treated agar surfaces were sampled using Rodac IRR LAB-Agar contact plates (BioMaxima S.A., Lublin, Poland) containing Sabouraud Dextrose Agar supplemented with chloramphenicol (Figure 3a,b). Each contact plate (55 mm diameter) was gently pressed against the irradiated area for 10 s to obtain a surface impression. The plates were then incubated aerobically at 37 °C for 24 h to allow visualization of any residual viable colonies.

#### 2.8.4. Image Acquisition and Quantitative Analysis

Post-incubation, digital images were captured under standardized white light conditions using a Nikon D70s DSLR camera (Nikon Corporation, Tokyo, Japan) equipped with an 18–70 mm f/3.5–4.5 NIKKOR lens positioned at a fixed 34 cm distance. Image analysis was conducted using ImageJ-Fiji software to quantify the remaining viable covered area. The percentage of surface coverage was calculated relative to the total irradiated zone for each microorganism and energy setting. This allowed for comparative evaluation of laser-induced reduction across species and energy levels.

### 2.9. Statistical Analysis

Two-way ANOVA was used to evaluate the effects of laser energy and microorganism type on inhibition zone diameter and viable coverage. Assumptions for parametric analysis were verified using the Shapiro–Wilk test for normality and Levene’s test for homogeneity of variances. When significant main effects were detected, Tukey’s HSD post hoc test was applied for pairwise comparisons between successive energy levels and among microbial species. Data are expressed as mean ± standard deviation. Statistical analysis was performed using STATISTICA software, version 13.0 (StatSoft, Tulsa, OK, USA), with significance set at *p* < 0.05.

## 3. Results

### 3.1. Evaluation of the Efficacy of the Er:YAG Laser in Inhibiting the Growth of Single-Species Cultures

**Figure 4:**
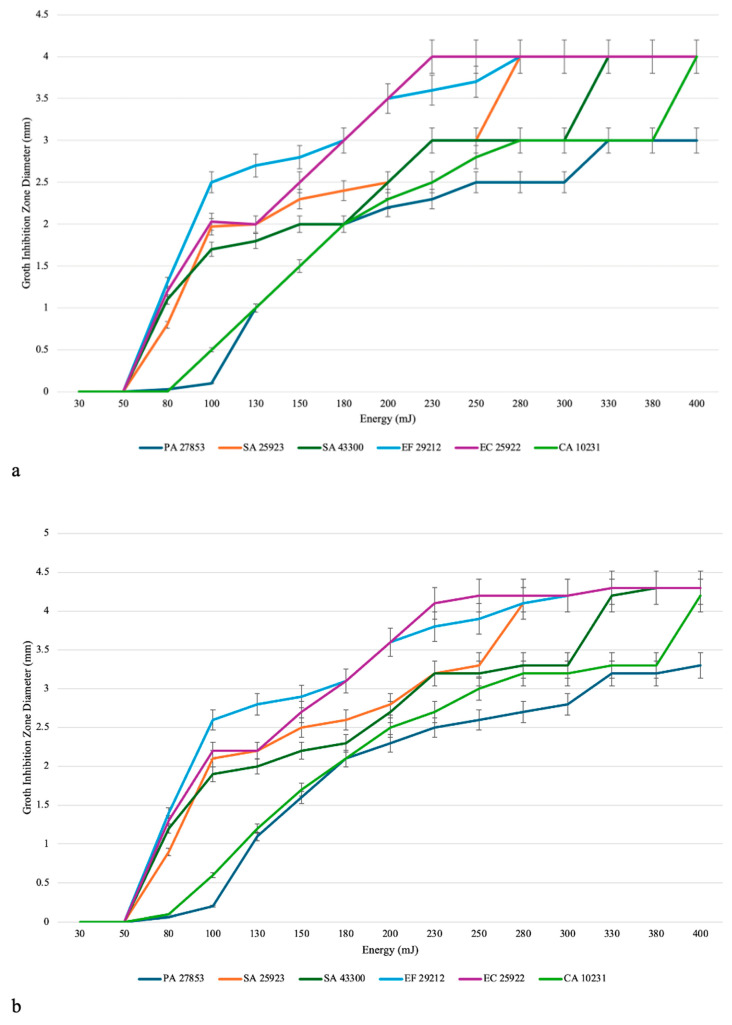
Comparative analysis of the antimicrobial activity of Er:YAG laser irradiation using two different laser tips. (**a**) Growth inhibition zone diameters (mm) for six single-species cultures, *Pseudomonas aeruginosa* (PA 27853), *Staphylococcus aureus* (SA 25923 and SA 43300), *Enterococcus faecalis* (EF 29212), *Escherichia coli* (EC 25922), and *Candida albicans* (CA 10231), irradiated with a tapered tip across increasing energy levels (30–400 mJ). (**b**) Corresponding results obtained using a flat tip under identical conditions.

Laser irradiation produced visible growth inhibition zones (GIZs) on all agar plates inoculated with *Pseudomonas aeruginosa* (PA 27853), *Staphylococcus aureus* (SA 25923 and SA 43300), *Enterococcus faecalis* (EF 29212), *Escherichia coli* (EC 25922), and *Candida albicans* (CA 10231). The inhibitory effects were detected at all tested energy levels (30–400 mJ) and became clearly visible 24 h after incubation. In contrast, non-irradiated control regions exhibited uniform, confluent microbial growth throughout the 96 h observation period. A progressive, energy-dependent increase in inhibition zone diameter was observed for all tested species (Figure 4). The relationship between laser energy and inhibition zone size demonstrated a positive correlation, confirming a dose-dependent antimicrobial effect. However, the magnitude of inhibition varied by organism, reflecting species-specific sensitivity to Er:YAG laser exposure. Among the tested species, *E. coli* (EC 25922) and *E. faecalis* (EF 29212) exhibited the largest inhibition zones, reaching approximately 4.0–4.3 mm at 230–250 mJ. *S. aureus* strains (SA 25923 and SA 43300) demonstrated intermediate responses, plateauing at around 3.5–4.0 mm. The smallest inhibition zones were recorded for *P. aeruginosa* (PA 27853) and *C. albicans* (CA 10231), which plateaued near 3.0–3.5 mm at the highest energy settings. This is presented in Figure 4.

**Figure 5:**
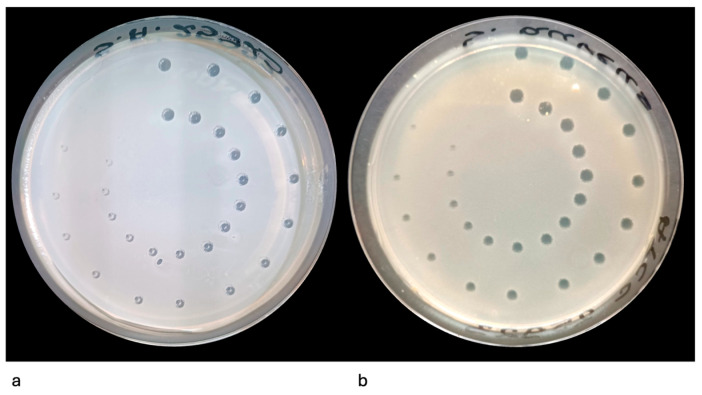
Er:YAG laser irradiation patterns obtained using two different fiber tip types. (**a**) Inhibition zones produced with the tapered tip (**b**) Inhibition zones generated with the flat tip under identical irradiation parameters.

When comparing the two optical laser tips, both configurations produced similar overall dose–response profiles. The tapered tip yielded slightly broader and more uniform inhibition zones, indicating wider lateral energy dispersion across the agar surface, whereas the flat tip produced sharper, more localized ablation sites consistent with a more focused beam profile (Figure 5). Statistical analysis (two-way ANOVA) confirmed significant effects of both laser energy (*p* < 0.001) and microorganism type (*p* < 0.001) on inhibition zone diameter. The interaction effect between energy and organism was not significant (*p* = 0.92), indicating that all tested species followed comparable inhibition trends as energy increased. These findings demonstrate that Er:YAG laser irradiation effectively inhibited microbial growth across all species in a power-dependent manner, with optimal disinfection occurring between 180 and 250 mJ.

### 3.2. Evaluation of the Efficacy of the Er:YAG Laser in Eliminating Mature Single-Species Agar Culture

**Figure 6:**
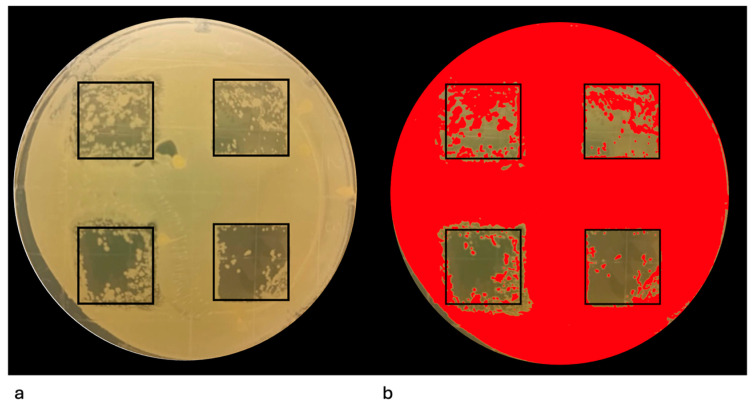
The experimental procedure used to quantify the percentage of surface area covered by single-species culture following Er:YAG laser irradiation. (**a**) Visible microbial growth within the marked areas indicates residual culture following treatment. (**b**) Processed image generated in ImageJ-Fiji software used for quantitative analysis. The red overlay represents the area covered by residual microbial colonies. This method allowed objective calculation of the percentage of coverage relative to the total irradiated surface for each tested microorganism.

The second phase assessed the ability of the Er:YAG laser to eliminate mature, 96 h-old single-species agar culture using four energy levels. Figure 6a shows representative Petri dishes with four defined irradiation zones corresponding to the applied energy settings. Figure 6b illustrates the ImageJ-Fiji analysis used to quantify the percentage of remaining viable culture. The red overlay indicates the analyzed surface area of remaining colonies, while the uncovered regions represent areas where colonies were eradicated successfully.

**Figure 7:**
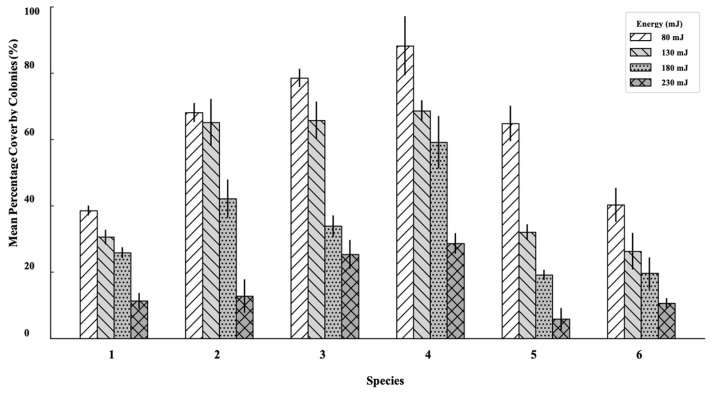
Effect of Er:YAG laser energy on the mean percentage surface coverage of viable coverage for six single-species cultures. The bar chart shows the mean (±SD) percentage of surface area covered by surviving colonies after laser irradiation at four different energy levels (80, 130, 180, and 230 mJ). Error bars represent the standard deviation. (1) *Escherichia coli* ATCC 25922 (2) *Staphylococcus aureus* ATCC 25923 (MSSA) (3) *Staphylococcus aureus* ATCC 43300 (MRSA) (4) *Enterococcus faecalis* ATCC 29212 (5) *Pseudomonas aeruginosa* ATCC 27853 (6) *Candida albicans* ATCC 10231.

Er:YAG laser irradiation induces a clear, energy-dependent reduction in viable coverage across both bacterial and fungal species. At the lowest energy level (80 mJ), most microorganisms maintained relatively high biomass density, with mean viable coverage exceeding 60–85% in *E. faecalis*, *P. aeruginosa*, *MRSA*, *MSSA* and *C. albicans,* exhibiting moderate coverage around 60–70% and 40%, respectively, while *E. coli* showed lower coverage at approximately 40%, indicating early susceptibility even at minimal exposure. When the energy was increased to 130 mJ, visible thinning and partial detachment occurred in all species, reflected by a reduction in viable surface coverage to 25–70%, depending on microbial type. The most substantial decreases were noted in *E. coli* and *P. aeruginosa*, suggesting a greater response to moderate irradiation. At 180 mJ, further declines were observed in all groups. *E. coli* and *S. aureus* ATCC 25923 fell below 30%, while *C. albicans* and MRSA retained 20–35% coverage. In contrast, *E. faecalis* and *P. aeruginosa* remained more resistant, maintaining 40–60% viable colonies. At the highest energy level (230 mJ), extensive eradication was observed for all species. The strongest effect occurred on *E. coli*, with residual coverage near 10%, followed by *S. aureus* (both strains) and *C. albicans*, which showed mean values around 10–15%. *E. faecalis* and *P. aeruginosa* demonstrated greater tolerance, retaining approximately 25–30% coverage (Figure 7).

The greatest reduction was observed in *E. coli* (ATCC 25922), where mean viable surface coverage decreased from 38.60 ± 1.46% at 80 mJ to 11.33 ± 2.40% at 230 mJ. *MSSA* exhibited a similar pattern, declining from 68.13 ± 2.92% to 12.80 ± 5.03%**,** while MRSA decreased from 78.57 ± 2.70% to 25.37 ± 4.32%. *C. albicans* (ATCC 10231) showed significant reduction from 40.33 ± 5.13% to 10.67 ± 1.53%. More resistant species, notably *P. aeruginosa* (ATCC 27853) and *E. faecalis* (ATCC 29212), required higher energies for meaningful reduction, maintaining residual viable coverage above 25% even at 230 mJ. Statistical analysis confirmed a significant influence of laser energy on reduction across all species (*p* < 0.05). Pairwise comparisons revealed significant differences between 80–130 mJ and 130–180 mJ (*p* < 0.05), while increases from 180 to 230 mJ produced smaller, though still measurable, improvements for most microorganisms.

These findings confirm that Er:YAG laser exposure exerts a measurable and energy-dependent elimination effect. However, the threshold for optimal efficacy varies by species, emphasizing the necessity of tailoring laser parameters to the biological characteristics and resistance profiles of different microorganisms.

### 3.3. Finding the Optimal Parameters for the In Vitro Eradication of Each Species

Based on the results of this in vitro study, the optimal Er:YAG laser parameters for effective inhibition and elimination of single-species agar-based cultures varied slightly among microorganisms but consistently achieved maximal antimicrobial activity at higher energy settings. During the inhibition-phase experiments (1 Hz frequency, 300 µs pulse duration, 10 mm distance, no air or water cooling), all species exhibited a clear, energy-dependent increase in inhibition zone diameter, with optimal effects occurring between 200 and 250 mJ. *Escherichia coli* ATCC 25922 and *Enterococcus faecalis* ATCC 29212 produced the largest inhibition zones (approximately 4.0–4.3 mm), while *Staphylococcus aureus* ATCC 25923 and *S. aureus* ATCC 43300 (MRSA) showed moderate inhibition (3.5–4.0 mm). *Pseudomonas aeruginosa* ATCC 27853 and *Candida albicans* ATCC 10231 required higher energy levels (230–250 mJ) to reach comparable inhibition (3.0–3.5 mm).

In the mature culture removal phase (10 Hz frequency, 300 µs pulse duration, contact mode at ~45°, 180 s irradiation per 12 × 12 mm zone, no air or water cooling), all species demonstrated progressive, energy-dependent decreases in viable surface coverage, with the most pronounced reductions observed at 230 mJ. *E. coli* ATCC 25922 showed the highest sensitivity, with mean surface coverage reduced from 38.60 ± 1.46% at 80 mJ to 11.33 ± 2.40% at 230 mJ. *S. aureus* ATCC 25923 and *S. aureus* ATCC 43300 (MRSA) followed similar patterns, declining to 12.80 ± 5.03% and 25.37 ± 4.32%, respectively. *C. albicans* ATCC 10231 exhibited notable susceptibility as well, decreasing from 40.33 ± 5.13% to 10.67 ± 1.53%. Conversely, *P. aeruginosa* ATCC 27853 and *E. faecalis* ATCC 29212 were the most resistant species, maintaining 19.17 ± 1.57% and 28.67 ± 3.06% viable coverage even at the highest tested energy. Although effective against all tested microorganisms, *E. faecalis* and *P. aeruginosa* demonstrated the greatest tolerance, suggesting the potential need for multiple passes, longer exposure, or adjunctive treatments to achieve complete eradication.

## 4. Discussion

### 4.1. Results in the Context of Evidence

The present study demonstrated that Er:YAG laser irradiation exerts a distinct, energy-dependent antimicrobial effect against the tested microbial species, supporting its potential as a targeted, non-pharmacological approach for reducing surface microbial growth. The observed positive correlation between laser energy and inhibition zone diameter across all tested species reflects the combined photothermal and photomechanical mechanisms through which Er:YAG radiation disrupts cells on hydrated surface material. Although this study was conducted under controlled in vitro conditions, the results establish a foundational understanding of how energy modulation influences microbial response to Er:YAG irradiation and support further species-specific parameter optimization.

Previous studies have found that the Er:YAG laser effectively inhibited the growth and promoted the elimination of mature single-species *Candida* surface growth in vitro, showing significant disinfection properties even at low energy doses, with the most effective results observed at 150 mJ [20]. Folwaczny et al. demonstrated that Er:YAG irradiation at 2.94 μm significantly reduced the bacterial load of *Escherichia coli*, *Staphylococcus aureus*, *Actinobacillus actinomycetemcomitans*, *Eikenella corrodens*, and *Peptostreptococcus micros* on root surfaces, with greater reductions observed at higher pulse counts, supporting a clear energy-dependent effect and species-specific variability in susceptibility [26]. Meire et al. further showed that Er:YAG irradiation in single-pulse mode achieved total inhibition thresholds (TITs) for *Enterococcus faecalis* and *Candida albicans* between 100 and 210 mJ, with higher energy pulses required for more resistant species, and that Er:YAG was superior to Nd:YAG for microbial inactivation on agar surfaces [27]. Noiri et al. found that Er:YAG irradiation effectively reduced viable cell counts and altered the morphology of multiple endodontic pathogens, including *E. faecalis*, with evidence of photothermal and photomechanical damage [28].

Additionally, Valenti et al. reported that Er:YAG treatment of carious lesions in vivo resulted in a 90–100 percent reduction in total microbial load, including *Streptococcus* and *Lactobacillus* species, compared to conventional therapy [29]. Randomized controlled trials and systematic reviews directly comparing Er:YAG laser therapy to conventional antimicrobial or mechanical procedures consistently show that Er:YAG laser therapy yields similar short-term clinical outcomes to scaling and root planing (SRP), with no significant differences in probing depth reduction, clinical attachment level gain, or bleeding indices at 3–6 months follow-up [33,34,35,36,37,38,39,40]. For deeper lesions (≥7 mm), Er:YAG may be as effective as SRP, and in some studies, adjunctive use with SRP provides marginal additional benefit, though the clinical significance is limited [37,38,39,40]. Safety profiles are comparable, with the Er:YAG laser associated with less postoperative sensitivity and similar rates of adverse events to conventional therapy [2,6]. Patient-reported outcomes, such as comfort and procedure duration, may favor Er:YAG therapy, which is often faster and perceived as less painful [2]. Evidence for fungal surface growth removal (e.g., *Candida* spp.) also supports Er:YAG irradiation as effective, especially when combined with chemical agents, though standardized protocols are still being refined [40,41,42,43,44].

### 4.2. Clinical Relevance and Broader Translation

Because the present model was limited to single-species surface growth on hydrated agar, it does not replicate the structural complexity or ecological characteristics of microbial communities found in clinical environments. Consequently, the findings should not be interpreted as direct evidence for clinical performance. Instead, the value of this work lies in its contribution to preclinical parameter optimization. Er:YAG irradiation has been investigated across various dental and medical contexts for its ability to reduce microbial load [33,34,35,36,37,38,39,40], and studies on root surfaces, endodontic pathogens, and carious lesions have documented its capacity to alter or inactivate microorganisms under controlled conditions [17,18,26,27,28,29]. The species-specific reference data generated here provide a standardized framework for understanding how different microorganisms respond to defined Er:YAG settings. These quantitative insights can be used to inform the design of future in vitro models, support cross-study comparison, and contribute to the methodological groundwork needed before evaluating Er:YAG parameters in more complex biological systems. Additional research involving mixed-species systems, in vivo validation, and assessment of thermal safety thresholds on biological substrates is essential to translate these findings into broadly applicable protocols.

### 4.3. Limitations of the Study

This investigation was performed under controlled in vitro conditions, which do not replicate the complex environment of the oral cavity, including saliva flow, crevicular fluid, host immune factors, and the presence of dental or implant surfaces that may influence laser energy absorption and heat dissipation [8,9]. The study examined only single-species agar-based surface cultures, whereas microbial communities in clinical settings are typically polymicrobial and structurally heterogeneous, with interactions that can alter tolerance to physical or thermal stress [20,30,31,32]. The microorganisms used were reference ATCC strains, and their responses may not fully reflect the phenotypic variability, virulence, or resistance traits found in contemporary clinical isolates. It should also be noted that the effects observed here may be influenced by the simplified experimental model. Laser exposure was applied to hydrated agar without air or water cooling, conditions that may have facilitated greater surface heating and more efficient disruption than would occur on natural dentin, titanium, or soft tissue substrates [19,20,26]. Likewise, the uniform and relatively thin surface layers produced under laboratory conditions likely permitted deeper laser penetration than would be expected in thicker or more irregular microbial accumulations. In more complex systems, higher energies, repeated passes, or adjunctive treatments may be necessary to maximize microbial reduction [21,22,23,24,27,28]. The sample size was suitable for preliminary optimization, but larger datasets would strengthen dose-response modeling and comparisons between laser tip geometries. Finally, real-time temperature changes within the substrate were not recorded, so potential thermal rises associated with contact mode Er:YAG delivery could not be evaluated. Future studies should integrate temperature monitoring on dentin, titanium, or soft tissue analogs to establish safe operating limits [17,18,24].

### 4.4. Significance of the Study

This study provides quantitative, species-specific reference data on how Er:YAG laser energy influences the reduction in single-species agar-based surface growth. By using a controlled in vitro model, it was possible to characterize clear, energy-dependent trends in both early growth inhibition and removal of mature surface layers across a diverse microorganism. The consistent correlation between higher pulse energies and greater microbial eradication supports previous findings that Er:YAG irradiation is capable of disrupting cells on hydrated substances [23,24,25]. The inclusion of multiple species under identical conditions highlights that susceptibility is not uniform. These differences illustrate the importance of species-dependent parameter selection when evaluating laser-based approaches. The comparison of the tapered and flat tips suggests that beam divergence and energy spread may play a role in determining surface-level reduction efficiency. Identifying a repeatable high-performing setting (230 mJ, 10 Hz, 300 µs, 180 s, contact mode) provides a practical benchmark for subsequent laboratory studies wishing to evaluate laser-mediated microbial reduction under standardized conditions. However, in clinical contexts these parameters must be adapted to the specific tissue conditions, since applying high energies without adjustment may increase the risk of thermal injury.

### 4.5. Clinical Translation

Although the present study provides detailed energy–response data, it is important to emphasize that the experimental model was limited to single-species agar-based growth on hydrated agar. This system does not capture the structural, ecological, or substrate-dependent characteristics of microbial communities found in clinical environments. For this reason, direct clinical extrapolation is not appropriate, and the results should be interpreted strictly as preclinical reference data rather than evidence of therapeutic performance. The value of the findings lies in their ability to inform the next stages of laboratory research. By identifying species-specific differences in susceptibility and defining energy thresholds that consistently reduce surface-associated microbial growth, the study establishes a quantitative basis for designing more advanced in vitro models. These may include multispecies systems, alternative substrate materials, or configurations that better simulate the physical properties of biological tissues. The data also provide a practical starting point for researchers seeking to select initial Er:YAG parameters for mechanistic studies on microbial reduction, energy distribution, or substrate interaction. The comparison between tapered and flat delivery tips offers additional methodological guidance. The broader energy spread produced by the tapered tip and the more concentrated pattern observed with the flat tip illustrate how optical geometry influences the distribution of energy across the surface. These observations can assist in choosing tip configurations that match specific experimental goals without implying any clinical preference.

## 5. Conclusions

This study systematically evaluated the effects of Er:YAG laser irradiation on single-species agar-based surface cultures and demonstrated clear reductions in both early growth and mature surface coverage. Species-specific variability was evident, with *E. coli* and *S. aureus* responding at lower or intermediate energies, while *P. aeruginosa* and *E. faecalis* required higher settings to achieve comparable reductions. The identification of a reproducible high-performing parameter set offers a practical reference point for subsequent laboratory work. The comparison of tapered and flat delivery tips further showed that optical geometry influences the effectiveness of single-species agar-based culture eradication. Although the experimental model does not replicate clinical microbial ecology, the findings provide quantitative baseline data that can guide the design of more advanced in vitro studies and support the rational selection of Er:YAG parameters in future research.

## Data Availability

All new data generated in this study is included in this manuscript.

## Abbreviations

The following abbreviations are used in this manuscript:

Er:YAG	Erbium-doped yttrium aluminum garnet
Nd:YAG	Neodymium-doped yttrium aluminum garnet
EPS	Extracellular polymeric substance
CFU	Colony-forming unit
MRSA	Methicillin-resistant *Staphylococcus aureus*
MSSA	Methicillin-sensitive *Staphylococcus aureus*
ATCC	American Type Culture Collection
TSA	Tryptic Soy Agar
SDA	Sabouraud Dextrose Agar
NaCl	Sodium chloride
RCT	Randomized controlled trial
ANOVA	Analysis of variance
SD	Standard deviation
GIZ	Growth inhibition zone
PA	*Pseudomonas aeruginosa*
SA	*Staphylococcus aureus*
EF	*Enterococcus faecalis*
EC	*Escherichia coli*
CA	*Candida albicans*
